# The World Health Organization 2030 goals for onchocerciasis: Insights and perspectives from mathematical modelling: NTD Modelling Consortium Onchocerciasis Group

**DOI:** 10.12688/gatesopenres.13067.1

**Published:** 2019-09-26

**Authors:** NTD Modelling Consortium Onchocerciasis Group

**Keywords:** onchocerciasis, elimination of transmission, mass drug administration, ivermectin, alternative treatment strategies, EPIONCHO, ONCHOSIM, NTD Modelling Consortium

## Abstract

The World Health Organization (WHO) has embarked on a consultation process to refine the 2030 goals for priority neglected tropical diseases (NTDs), onchocerciasis among them. Current goals include elimination of transmission (EOT) by 2020 in Latin America, Yemen and selected African countries. The new goals propose that, by 2030, EOT be verified in 10 countries; mass drug administration (MDA) with ivermectin be stopped in at least one focus in 34 countries; and that the proportion of the population no longer in need of MDA be equal or greater than 25%, 50%, 75% and 100% in at least 16, 14, 12, and 10 countries, respectively. The NTD Modelling Consortium onchocerciasis teams have used EPIONCHO and ONCHOSIM to provide modelling insights into these goals. EOT appears feasible in low-moderate endemic areas with long-term MDA at high coverage (≥75%), but uncertain in areas of higher endemicity, poor coverage and adherence, and where MDA has not yet, or only recently, started. Countries will have different proportions of their endemic areas classified according to these categories, and this distribution of pre-intervention prevalence and MDA duration and programmatic success will determine the feasibility of achieving the proposed MDA cessation goals. Highly endemic areas would benefit from switching to biannual or quarterly MDA and implementing vector control where possible (determining optimal frequency and duration of anti-vectorial interventions requires more research). Areas without loiasis that have not yet initiated MDA should implement biannual (preferably with moxidectin) or quarterly MDA from the start. Areas with loiasis not previously treated would benefit from implementing test-and(not)-treat-based interventions, vector control, and anti- *Wolbachia* therapies, but their success will depend on the levels of screening and coverage achieved and sustained. The diagnostic performance of IgG4 Ov16 serology for assessing EOT is currently uncertain. Verification of EOT requires novel diagnostics at the individual- and population-levels.

## Abbreviations

ABR, annual biting rate; APOC, African Programme for Onchocerciasis Control; ATS, alternative treatment strategies; DALY, disability-adjusted life-year; EMC, Erasmus Medical Center; EOT, elimination of transmission; EPHP, elimination as a public health problem; ICL, Imperial College London; IU, implementation unit; MAP, maximum *a posteriori* (parameter set); MDA, mass drug administration; NTD, neglected tropical disease; OCP, Onchocerciasis Control Programme in West Africa; PCR, polymerase chain reaction; pOTTIS, provisional operational thresholds for treatment interruption and initiation of surveillance; PPV, positive predictive value; SSA, sub-Saharan Africa; Ta(N)T, test-and-not-treat; TaT, test-and-treat; WHO, World Health Organization; YLD, years of life lost to disability.

## Disclaimer

The views and opinions expressed in this article are those of the authors and do not necessarily reflect those of the World Health Organization. Publication in Gates Open Research does not imply endorsement by the Gates Foundation.

## Background

Onchocerciasis is a filarial disease caused by infection with *Onchocerca volvulus*, a vector-borne parasitic nematode transmitted via the bites of several *Simulium* blackfly species. In sub-Saharan Africa (SSA), where 99% of the cases occur, the main vectors belong to the *Simulium damnosum sensu lato* species complex. Modelling of data from the 2013 Global Burden of Disease Study suggests that at least 17 million people are currently infected with *O. volvulus* ^1^. An estimated 198 million people live in areas where there is potential for transmission of the parasite, although this number may increase as the mapping of areas of low transmission is finalized ^2^. The disease is also known as river blindness because blackflies breed in and bite near fast-flowing rivers, and because the most devastating sequela is irreversible loss of vision. High infection load, measured by the density of microfilariae (the larval progeny of adult worms) in the skin, is associated both with blindness incidence ^3^ and excess human mortality, the latter over and above that due to blindness ^4, 5^. In addition to ocular sequelae, onchocerciasis is also responsible for skin disease and troublesome itching (which disturbs sleep and work patterns). The Global Burden of Disease Study 2015 estimated the disability-adjusted life years (DALYs) due to onchocerciasis as 1,136,000 in 2015 (a 21% decrease since 2005) ^6^. More recently, the term river epilepsy has been used to highlight the association between onchocerciasis and nodding syndrome (a condition in the epilepsy spectrum) ^7^. The burden of onchocerciasis-associated epilepsy for 2015 was estimated as circa 13% of the total years lost due to disability (YLDs) attributable to onchocerciasis and 10% of those attributable to epilepsy ^8^.

Currently, the mainstay of onchocerciasis control is through mass drug administration (MDA) of the microfilaricidal drug ivermectin (Mectizan®), donated by Merck & Co. Inc. to endemic countries through the Mectizan Donation Program ^9^. Ivermectin MDA (mostly of annual frequency in SSA) started in the late 1980’s, and great strides in reducing morbidity have been made ^10^. Although questions were raised concerning the feasibility of eradicability based on ivermectin MDA alone ^11– 13^, studies in some foci of Mali and Senegal (in the western extension of the Onchocerciasis Control Programme in West Africa, OCP), indicated that local elimination may be achieved after 15–17 years of ivermectin MDA ^14, 15^. Based on this, in 2010, the African Programme for Onchocerciasis Control (APOC) shifted its goals from elimination of the disease as a public health problem (EPHP), to elimination of transmission (EOT) ^16^. This shift necessitates a drastic geographical extension of treatment. Whilst for EPHP, mesoendemic and hyperendemic areas had been prioritized for ivermectin MDA, EOT at a Pan-African scale requires that treatment be distributed also in hypoendemic areas. This poses challenges, particularly where loiasis (a filariasis caused by *Loa loa*) is co-endemic due to severe adverse events, including fatalities, that may result from microfilaricidal treatment of individuals heavily infected with *L. loa* ^17^. In onchocerciasis, the original definition of endemicity levels comprises three categories: i) hypoendemic (microfilarial prevalence <30–35%), ii) mesoendemic (prevalence between 30–35% and 60%), and iii) hyperendemic (≥60%) ^18^; EOT should be achieved in all three. A microfilarial prevalence ≥80% has also been used to indicate holoendemicity ^19^.

EOT is formally defined as a reduction to zero in the incidence of infection (rate at which new cases arise) ^20^. However, since in macroparasite epidemiology the number of parasites in the population is more relevant than the number of cases, the definition of EOT used here is the absence of adult worms and larval stages (in humans and vectors) after cessation of interventions ^21^. Onchocerciasis is in the fortunate position that ivermectin reduces: i) morbidity (mainly caused by microfilariae), ii) microfilarial prevalent cases and microfilarial load, and iii) transmission to vectors (microfilariae are the stages infective to blackflies). This is achieved by a combination of ivermectin’s microfilaricidal effect, temporary embryostatic effect on adult female worms (macrofilariae) ^22^, and irreversible effects on female worm fecundity and/or survival ^23, 24^. Given the disease- and transmission-curtailing benefits of ivermectin MDA, the 2012 World Health Organization (WHO) roadmap to accelerate progress in combating neglected tropical diseases (NTDs) proposed that onchocerciasis be eliminated in selected African countries by 2020 ^25^. The Joint Action Forum of APOC reformulated this goal to EOT in 80% of endemic African countries by 2025 ^26^.

To help evaluate progress towards these goals, the developers of two mathematical models of onchocerciasis transmission dynamics and control (EPIONCHO and ONCHOSIM) have joined forces under the umbrella of the Bill & Melinda Gates Foundation-funded NTD Modelling Consortium. The modelling groups, based at Imperial College London (ICL), UK, and Erasmus Medical Center (EMC), The Netherlands, along with multi-disciplinary collaborators have led the recent onchocerciasis work. Firstly, a comparison of modelling assumptions and resulting outputs was conducted ^27^, highlighting the need to present and discuss the models jointly ^28^, and modify some key assumptions crucial to fitting the microfilarial prevalence trends observed in the elimination studies of Mali and Senegal ^29^. A policy paper followed, discussing the role of intervention strategies other than annual ivermectin MDA to achieve elimination in SSA ^30^. A further understanding of critical uncertainties led to a paper discussing these in light of data needs ^31^. Due to knowledge gaps surrounding the processes regulating parasite establishment and fecundity of *O. volvulus* ^32– 34^, the magnitude of exposure heterogeneity and its interaction with such regulatory processes ^31^, and the impact of treatment on population biology parameters, including the development of acquired immunity ^35^, the models have contrasting underlying assumptions leading to differences in model predictions ^29, 30^. Despite these differences, the models generally agree on the treatment strategies required to achieve EOT.

Moving towards the post-2020 goals, new NTD goals have been proposed by the WHO for 2030 within the framework of a consultation process. Table 1 summarizes the current (2020) and proposed (2030) goals for onchocerciasis, the scenarios in which both models agree that EOT is technically feasible with current interventions, the alternative treatment strategies (ATS ^36, 37^) required when current ones are not sufficient, the suitability of available tools for evaluating EOT, the principal uncertainties driving differences in model outputs, and the biggest risks associated with the proposed 2030 goals.

**Table 1.** Summary of modelling insights and challenges for reaching the World Health Organization (WHO) 2030 goals for onchocerciasis.

Current WHO Goal (2020 Goal)	Elimination of transmission (EOT) by 2020 in Latin America, Yemen, selected African countries.
Proposed New WHO Goal (2030 Goal)	Verified EOT in 10 countries; stopped mass drug administration (MDA) in at least one focus in 34 countries; stopped MDA in ≥ 25%, ≥50%, ≥75% and 100% of population in >16, >14, >12, >10 countries, respectively.
Is the new target technically feasible under the current intervention strategy?	EOT feasible in areas with low or moderate endemicity, where MDA has been ongoing for several years; uncertain for hyper- and holoendemic areas or where MDA has not yet started (distribution of pre-intervention prevalence in different countries will determine feasibility of stopping MDA goals).
If not, what is required to achieve the target? (updated strategy, use of new tools, etc.)	Hyper- and holoendemic areas: switch to biannual or quarterly MDA, complementary vector control; areas without loiasis not previously treated: biannual MDA, quarterly MDA, moxidectin. Areas with loiasis not previously treated: Ta(N)T *, vector control, TaT** with anti- *Wolbachia* therapies.
Are current tools able to reliably measure the target?	Sensitivity and specificity of standardized IgG4 antibody tests to Ov16 antigen for assessing EOT are uncertain. Diagnostic tools for detection of reproductively active adult worms are needed.
What are the biggest unknowns?	Whether parasite acquisition becomes more efficient with declining transmission intensity; age- and sex-dependence and individual heterogeneity in exposure; additional impact of vector control; dynamics of Ov16 antibody responses; human/vector movement.
What are the biggest risks?	EOT may not be feasible with current tools in hyper- and holoendemic areas; risk of resurgence if MDA is stopped prematurely; interruption/late commencement of MDA; population movement.

*Ta(N)T: Test-and-not-treat: test for *Loa loa* microfilaraemia load (e.g. with LoaScope, and if ≥20,000 microfilariae/ml blood (associated with severe adverse events following microfilaricidal treatment ^17^), not treat with ivermectin ^39^. TaT: Test-and-treat: test for *O. volvulus* (e.g. with skin snips) and if positive treat with anti- *Wolbachia* (e.g. doxycycline) therapy ( *L. loa* lacks *Wolbachia*).

## Insights gained from quantitative and mathematical modelling analyses

*1) Initial model comparison*: Predictions from EPIONCHO and ONCHOSIM were compared over a range of baseline endemicities and treatment scenarios. In particular, comparisons were made on: 1) microfilarial prevalence and intensity during 25 years of (annual or biannual) MDA with ivermectin; 2) required duration of treatment to bring microfilarial prevalence below a provisional operational threshold for treatment interruption (pOTTIS) of 1.4% ^16, 38^; and 3) required duration to drive the parasite population to local elimination (defined by stochastic fade-out in ONCHOSIM and passing the transmission breakpoint in EPIONCHO). In mesoendemic areas, the models predicted that the provisional operational prevalence threshold could be reached with annual MDA. In highly hyperendemic areas, the models indicated that annual MDA would be insufficient. In lower endemicity settings, ONCHOSIM predicted that the time needed to reach 1.4% microfilarial prevalence would be longer than that required to reach local elimination; the opposite was true for higher endemicity settings. In EPIONCHO, the pOTTIS was reached consistently sooner than the breakpoint ^27^.

**Figure 1.**
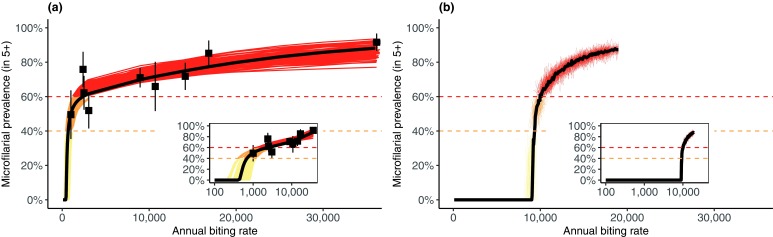
Modelled relationship between annual biting rate (ABR) and endemic microfilarial prevalence. In EPIONCHO ( **a**), the coupled ABR-prevalence data are from nine communities in northern Cameroon, with each ABR measured as an average from multiple years and locations within and around each community, weighted by the proportion of time community residents spent at these locations ^40^. Each thin line corresponds to an EPIONCHO parameter set identified by a sampling importance resampling procedure to account for parametric uncertainty. These are colored sequentially from yellow (hypoendemic) to red (hyperendemic). The thick black line corresponds to the parameter set that achieved the highest likelihood. In ONCHOSIM ( **b**), the thin lines correspond to stochastic realizations using the default parameter set ^21^, colored sequentially according to endemicity category; the thick black line is the median of 500 simulations. The coupled ABR-prevalence data are not shown in ( **b**) because ONCHOSIM has not been re-fitted to these data. ABR = No. blackfly bites/person/year. This figure has been reproduced from 29 under a Creative Commons Attribution-NonCommercial-NoDerivatives 4.0 International (CC BY-NC-ND 4.0) license.

**Figure 2.**
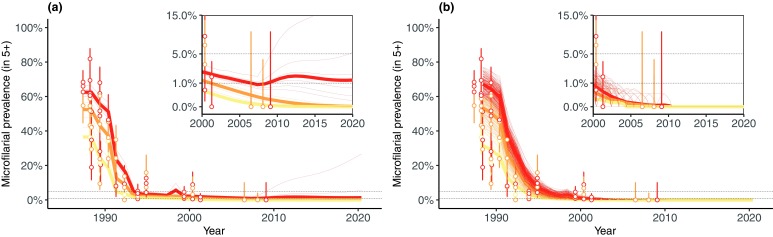
Observed and modelled microfilarial prevalence dynamics in 14 communities from the River Gambia focus, Senegal. Left ( **a**) and right ( **b**) panels show, respectively, EPIONCHO and ONCHOSIM projections. The thin lines correspond to community-specific simulations using maximum likelihood estimates of the community-specific ABRs and either the maximum *a posteriori* (MAP) parameter set (EPIONCHO) or the default parameter set (ONCHOSIM). The estimated ABRs and MAP parameter sets were derived using the complete longitudinal sequence of microfilarial prevalence for each community. For ONCHOSIM there are many stochastic projections for each community; for EPIONCHO there is a single deterministic projection for each community, corresponding to the MAP parameter set. The thick solid lines show the median dynamics by endemicity category (yellow: hypoendemic; orange: mesoendemic; red: hyperendemic). In the River Bakoye focus of Mali, no resurgence was predicted by either model using the entire longitudinal microfilarial prevalence set. Panel insets show the period between 2000 and 2020 using a transformed *y*-axis for a better visual appraisal of the model projections compared to the data close to zero. This figure has been reproduced from 29 under a Creative Commons Attribution-NonCommercial-NoDerivatives 4.0 International (CC BY-NC-ND 4.0) license.

*2) Modelling elimination studies*: Based on the initial model comparison, technical refinements were implemented (e.g. age-dependent adult worm mortality in EPIONCHO). EPIONCHO and ONCHOSIM projections were tested against microfilarial prevalence data from the two foci in Mali and Senegal where the observed prevalence had been brought to zero in 2007–2009 after 15–17 years of ivermectin MDA only ^14, 15^. Model projections were trained using longitudinal (microfilarial prevalence) data from 27 communities in two transmission foci, incorporating programmatic information (treatment frequency, duration and coverage), and evaluating whether the projected outcome was elimination (local parasite extinction) or resurgence. The epidemiological trends during MDA were captured by both models but resurgence was predicted by EPIONCHO in some communities of the River Gambia focus in Senegal with the highest (inferred) annual biting rates (no. vector bites/person/year) and associated pre-intervention endemicities ( Figure 1 and Figure 2) ^29^. Resurgence was never predicted by ONCHOSIM.

*3) Alternative treatment strategies*: ONCHOSIM and the refined version of EPIONCHO ^29^ were used to simulate trends in microfilarial prevalence for 80 different settings. These settings were defined by their baseline endemicity and past programmatic scenario (frequency and coverage of MDA) and future treatment scenarios defined by the frequency (annual, biannual, or quarterly), with or without vector control. Each strategy was assessed whether it eventually led to elimination ^30^. Both models predicted that in areas with 40%–50% pre-control microfilarial prevalence and ≥10 years of annual MDA, elimination may be achieved within a further 7 years without changing strategy. For areas with 70%–80% pre-control microfilarial prevalence, both annual and biannual MDA were predicted by ONCHOSIM (but not by EPIONCHO) to be sufficient strategies to reach elimination by 2025. The likelihood that elimination will be reached thus depends on pre-control endemicity (i.e. transmission intensity), duration and frequency of past MDA, and the strategy implemented from now until 2025. Biannual or quarterly MDA will accelerate progress toward elimination but it cannot be guaranteed by 2025 in high-endemicity areas. In such areas, MDA plus concomitant vector control would be useful ^30^.

**Figure 3.**
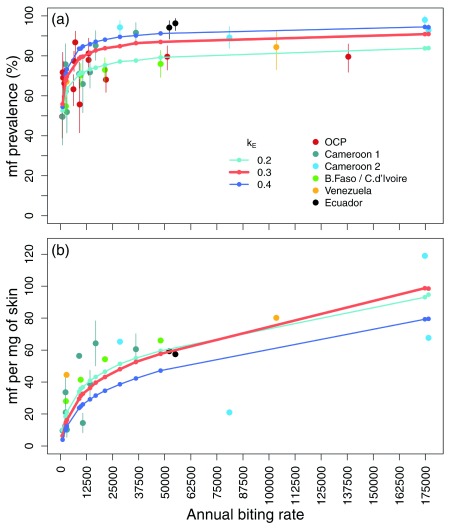
Observed and predicted pre-intervention microfilarial prevalence and intensity vs. annual biting rates (ABRs). EPIONCHO-IBM-predicted (solid lines) ( **a**) microfilarial prevalence (percent) and ( **b**) intensity (mean no. of microfilariae, mf, per mg of skin) for ABRs in the epidemiological dataset (solid color circles), using the estimated parameters. The overdispersion exposure parameter (of a gamma distribution) *k*_E_ was varied between 0.2 (stronger aggregation of blackfly bites among humans) and 0.4 (lesser aggregation). A value of *k*_E_ = 0.3 provided the best overall fit. Error bars are 95% confidence intervals (bootstrapped for intensity when raw data were available). Fitting data are from Cameroon [1] ^40^, [2] ^41^, Burkina Faso/Côte d’Ivoire ^42^; validation data are from the Onchocerciasis Control Programme in West Africa (OCP) ^43^, Venezuela ^44^ and Ecuador ^45^ (the latter two for vectors with similar vector competence to *S. damnosum sensu stricto*). This figure has been reproduced from 31 under a Creative Commons Attribution 4.0 International (CC BY 4.0) license.

*4) Resilience to interventions*: Both models incorporate microfilarial density-dependent establishment of *O. volvulus* L1 larvae within savannah species of *S. damnosum sensu lato* (i.e. *S. damnosum sensu stricto / S. sirbanum*) ^28^. However, only EPIONCHO assumes transmission intensity-dependent parasite establishment within humans ^31, 32^. An individual-based, stochastic version of EPIONCHO (EPIONCHO-IBM) was developed to explore the interaction between individual heterogeneity in exposure to infection (via vector bites) and parasite establishment within humans (greater at lower transmission intensities and vice versa). Transmission intensity is measured by the annual rate of human exposure to infective (L3) larvae (no. L3 larvae/person/year). EPIONCHO-IBM was fitted (using Latin hypercube sampling) to matched (savannah) pre-intervention microfilarial prevalence, load and annual biting rate (ABR) data (extending Figure 1a range). Density dependence in parasite establishment within humans was estimated for different levels of (fixed) exposure heterogeneity ( Figure 3) ^31^. The interaction between overdispersion in exposure to blackfly bites (parameter *k*_E_) and density-dependent parasite establishment stabilizes low (hypoendemic) prevalence, but accentuates resilience to MDA, explaining the lower EOT probabilities predicted by EPIONCHO.

In view of (1)–(4) above, Box 1 summarizes the insights gained thus far and their implications for post-2020 goals.

Box 1. **Lessons learned from onchocerciasis modelling and implications for the post-2020 goals.**

- ***Elimination of transmission (EOT) prospects depend strongly on local transmission conditions*.** The required duration of interventions increases and the probability of reaching EOT decreases with higher baseline endemicity, higher vector biting rates, and stronger aggregation of infection in the human host population (due to exposure heterogeneity) ^27, 29, 31^.
- ***Programme effectiveness is important*.** Program duration increases, and probability of EOT decreases, with lower coverage and higher systematic non-adherence ^46, 47^.
- ***Current strategies may not be sufficient*.** Implementation of current strategies (annual mass drug administration [MDA], with at least 65% coverage) would lead to long timelines to EOT in some countries. Even with the more optimistic model (ONCHOSIM), some countries would need to continue their programs until 2045 ^48^.
- ***Alternative treatment strategies (ATS) to accelerate EOT*.** a) Improve MDA coverage (to 80%); b) minimize systematic non-adherence; c) increase MDA frequency (to biannual or quarterly) ^30, 46^; d) use more efficacious treatment regimens (moxidectin ^49^, anti- *Wolbachia* drugs ^50^; e) where feasible, implement additional vector control (e.g. ground-based larviciding of vector breeding sites; slash-and-clear of vegetation substrates of vector immature stages; adult blackfly traps) ^30, 51, 52^.
- ***Key uncertainties*.** EPIONCHO and ONCHOSIM model outputs diverge concerning the feasibility of EOT in hyperendemic areas (60-80% microfilarial prevalence) with current or thus far modelled ATS strategies ^30^, reflecting uncertainty about key biological processes (e.g. those regulating parasite establishment within humans and exposure heterogeneity) ^31^. For holoendemic areas (80% prevalence and above), both models agree that current strategies will be insufficient.
- ***Expansion of interventions into (currently untreated) hypoendemic areas.*** Work is ongoing to define efficient sampling strategies for delineation of new (hypoendemic) treatment areas, based on serology-based start-MDA thresholds. This is problematic in areas where loiasis is co-endemic, as ivermectin treatment can be fatal in individuals with high *Loa loa* microfilaraemia ^17^. A test-(for loiasis)-and-not-treat (if microfilaraemia is above dangerous thresholds) strategy can be used ^39^. Because the proportion of people with heavy *L. loa* microfilaraemia is often small (~2–4%), models predict that this strategy could lead to EOT if screening and treatment coverage/adherence are high.

## Practical implications of the currently proposed EOT goals

### Measuring the target

1) Definition of EOT according to the models.

EOT is modelled as absence of parasites in humans and flies 50 years after treatment cessation ^21^. The probability of EOT is the proportion of runs out of the total number of simulations in which EOT is achieved. In practice, evaluating whether foci are in track to achieve EOT depends on the ability of current (and novel) diagnostics to measure accurately interruption of transmission. Indicators of exposure in children and fly samples have been suggested for such evaluation.

2) Diagnostics.

The WHO guidelines for verification of EOT require <0.1% IgG4 antibody seropositivity to the Ov-16 *O. volvulus* antigen in children younger than 10 years, and <0.05% positivity by pool screen PCR in at least 6,000 wild-caught flies (heads only) ^53^. Models suggest that the magnitude of the serological thresholds will depend on: i) local transmission conditions (e.g., the higher the ABR, the lower the thresholds will need to be and vice versa; this also applies to entomological indicators); ii) age-dependent exposure patterns (e.g. the younger the age at which exposure to transmission starts, and the greater the rate of increase with age, the younger the age group for which serological monitoring will be informative and vice versa; see below for sampling implications); iii) history of previous control interventions; iv) heterogeneity concerning the above within evaluation areas; v) spatial scale at which EOT is assessed; vi) desired positive predictive value (PPV, the probability of elimination below the said threshold) of EOT; vii) assumptions on parasite population regulation; and viii) diagnostic performance of the tests ^54^.

3) Sources of uncertainty in current threshold estimates.

A key uncertainty is the operation of regulatory processes upon within-human parasite establishment (e.g. due to acquisition of anti-L3 immunity in areas of intense transmission). Omitting such processes (ONCHOSIM) leads to higher (more lenient) thresholds; including them (EPIONCHO) results in lower (more stringent) thresholds. Adjusting modelling results by the diagnostic performance of the tests changes the apparent magnitude of the thresholds. Diagnostic performance (e.g. sensitivity/specificity) of current tests is another key uncertainty ^54^.

4) Sampling implications.

Modelling indicates that the childhood age group most informative for Ov-16 serology will depend on the age-specific patterns of exposure that prevail in the various foci. In areas where exposure increases quickly from birth, the 0–9yr age group may be suitable. In areas where exposure increases more slowly, the 5–14yr age group will be more informative. Deciding which age group to choose can be aided by inspecting baseline infection age profiles ^55^.

### Timeline to achieve the target

1) Technical feasibility

- **General considerations**. Elimination prospects depend strongly on local transmission conditions ( Box 1). The required strategy intensifies (e.g. the duration and/or frequency of interventions increases, and the need for complementary/ATS increases) with higher baseline endemicity, higher ABRs, and stronger aggregation of infection in the human population (acting as a core group maintaining transmission) ^13, 27, 31^.
- **Moderate transmission settings**. EOT can be achieved with annual or biannual MDA if coverage and adherence are high (in the models, ‘enhanced’ coverage is defined as 80% therapeutic coverage and 1% systematic non-adherence).
- **Hyper- and holoendemic settings**. The model-predicted feasibility of EOT is more uncertain, even when modelling the ATS considered thus far (increased coverage and frequency, vector control ^30^). The epidemiological impact of other ATS (e.g. moxidectin ^49^, macrofilaricides ^50, 56^) needs to be evaluated.

2) Operational feasibility

- **General considerations.** Multiple intervention strategies will need to be combined in an optimal way to achieve continent-wide EOT. Further research is needed to identify such strategies according to epidemiological features ^30^.
- **Role of epidemiology/transmission ecology**. The choice of intervention strategies will depend on epidemiological and ecological features. Hyperendemic areas would benefit from biannual/quarterly MDA if feasible to implement (non-loiasis co-endemic ^57, 58^; no sub-optimal responses to ivermectin ^59^); highly hyperendemic/holoendemic areas will likely need optimized ATS, including macrofilaricidal interventions ^36, 37^. The optimal ATS suite (vector control, moxidectin, anti- *Wolbachia* therapies, macrofilaricides) needs to be determined according to setting. Programs need to expand into currently untreated hypoendemic areas, including untreated loiasis co-endemic areas ^58^ (see above for test-and(not)treat strategies ^39^).
- **Programmatic features**. Achieving and maintaining high coverage and adherence over each MDA round is paramount, especially in highly endemic settings. Long-term treatment can suffer from programme, communities and donor fatigue. Weak EOT programs need strengthening ^60^.

3) Ability to sustain achievement of the goal

- **Dynamics of resurgence**. The dynamics of resurgence according to setting need further investigation ^29^, as well as the factors leading to resurgence, e.g. residual (endogenous) infections or re-introduction of (allogenous) infections from surrounding areas through migration of humans and/or flies. Investigation of these dynamics will inform post-treatment and post-elimination surveillance protocols ^53^.

4) Considerations of cost

- **Health and economic benefits.** Achieving EOT in Africa would lead to substantial health and economic benefits, reducing the needs for health workforce and outpatient services ^61^. ATS needs and cost-effectiveness remain to be captured ^37, 49, 62, 63^.
- **Logistic and cost implications**. Striving for Pan-African EOT has considerable logistic and cost implications, due to required programme expansion into currently untreated areas, prospects of enhanced or more frequent MDA or ATS, and improved (post-treatment and post-EOT) surveillance. The increased costs should be weighed against shorter programme duration and higher probability of success. Costs per year are higher for biannual than for annual MDA, but costs do not simply double ^38, 63^. Annual moxidectin may be more cost-effective than biannual ivermectin, should this drug be donated ^49^. Economic analyses of moxidectin, anti- *Wolbachia* therapies, macrofilaricides, and vector control (ground larviciding, slash-and-clear, adult fly traps) need to be conducted. The costs of test-and-not-treat for loiasis co-endemic areas were recently assessed in Cameroon ^62^.

## Risks faced by treatment programs that need to be mitigated to achieve EOT

Some programs, particularly those in areas affected by conflict are more likely to under-perform, suffer from interrupted MDA schedules, or be delayed in starting and up-scaling implementation. Internal population displacement as well as mass migration to other countries could jeopardize progress towards EOT. Weak programs/health systems need to be identified, supported and strengthened ^60^. Movement of infected individuals (between endemic communities within countries, and between-countries) due to traditional cultural and trade patterns may lead to re-introduction of infection in areas achieving EOT. Risk of declining drug efficacy following multiple rounds of MDA/existence of sub-optimal responses to treatment should be monitored ^59, 64^. Low coverage and systematic non-adherence may lead to persistent transmission, particularly (but not exclusively) in loiasis co-endemic areas ^65^. Factors associated with non-compliance need further investigation. Reported coverage levels are generally higher than true coverage, possibly because coverage has been a primary indicator of program effectiveness. Population denominators may not be correct due to lack of updated censuses. Reported coverages are aggregated by district, but disaggregate data are needed to evaluate and correct coverage heterogeneity.

## Modelling priorities to support goals in the 2030 horizon and beyond

Table 2 outlines the priority modelling questions for further research that were elaborated in discussion with the WHO.

**Table 2.** Modelling priorities for further work discussed with the World Health Organization (WHO).

Priority issue / question identified in discussion with WHO	How can quantitative and mathematical modelling address this?
1. Assess time to elimination of transmission (EOT) based on ivermectin mass drug administration (MDA)	1.1 Generate projections, by implementation unit (IU), of trends in infection since the start of MDA, using both models. Identify countries that are likely to achieve country- wide EOT by 2030 or beyond. 1.2 Identify IUs that are expected to be able to stop MDA by 2023, 2025, 2030. Estimate the endemic population size by IU and estimate the % of the endemic population no longer requiring MDA by 2023, 2025, 2030 for each country.
2. Estimate elimination thresholds with uncertainty boundaries for different infection indicators at community level	2.1 Simulate serological (and potentially entomological) thresholds for elimination. First estimates have already been published for ONCHOSIM ^54, 66^. Compare these to estimates for EPIONCHO-IBM to understand their dependency on structural uncertainties and assumptions about local transmission conditions. 2.2. Identify optimal demographic groups for serological monitoring.
3. Assess potential for resurgence when MDA is stopped based on current/revised decision algorithms and stopping criteria	3.1 Revise current (serological and entomological) stopping criteria based on (2) above. 3.2 Investigate dynamics of recrudescence following cessation of MDA with both models.
4. Evaluate the potential of macrofilaricides as alternative treatment strategies to accelerate EOT	4.1 Generate projections of the epidemiological impact of macrofilaricides. 4.2 Conduct cost-effectiveness analyses.

## Data availability

No data are associated with this article.
