# Supplementary material for: The World Health Organization 2030 goals for onchocerciasis: Insights and perspectives from mathematical modelling: NTD Modelling Consortium Onchocerciasis Group
Source: Gates Open Res. 2019 Sep 26;3:1545. [Version 1] doi: 10.12688/gatesopenres.13067.1 (PMC6820451; doi:10.12688/gatesopenres.13067.1)
Supplement: Peer review history

# Peer review: Reviewer response for version 1

**DOI:** 10.21956/gatesopenres.14198.r28086

**Published:** 2019-10-31

**Authors:** Kenneth Pfarr

**Affiliations:** 1 Institute of Medical Microbiology, Immunology and Parasitology, University Hospital Bonn, Bonn, Germany

The authors have submitted an open letter summarizing modelling with EPIONCHO and ONCHOSIM to determine if and under what conditions elimination of transmission of onchocerciasis, as proposed by the WHO by 2030, can be achieved. The discussion of the previously published results focused on four aspects of quantitative and mathematical modelling analyses: initial model comparison, modelling elimination studies, alternative treatment strategies, and resilience to interventions to define lessons learned and implications for the goals.

 Both models predict that elimination of transmission is possible by the WHO proposed dates in areas that have already started MDA and where coverage is at least 75%, which is higher than the currently accepted 65% coverage. Hyperendemic regions and those regions that have only just recently started or have not started will require biannual to quarterly MDA to keep to this schedule. Areas coendemic with loiasis could also achieve elimination using test-and-not-treat and alternative strategies, e.g. anti- *Wolbachia* treatments. However, the latter will need newer analysis once results from treatment in such areas become available.

 The analysis of the results from both models highlighted the need for greater research into diagnostics of onchocerciasis infections and the biology of infection, especially in how it relates to microfilariae prevalence and transmission rates. Both areas are sources of uncertainty in the modelling. Nevertheless, the authors provided straight forward conclusions as to the practical implications of the current elimination goals and timeline to achieving them, and provide a list of priorities discussed with the WHO for prioritizing modelling in support of the goals by 2030 and beyond.

 To improve the manuscript, I have the following suggestions for changes:

1. Figures 1 and 2, define “(in 5+)” in the y-axes for those without access to the original publication.
2. Figure 1 legend, define panel insets as was done in Figure 2.
3. To help set the magnitude, it would be helpful to know the number of countries/foci that are in the different endemic categories: hypo-, meso-, hyper- and holoendemic. I believe this will be useful in highlighting where elimination can be achieved using current MDA strategies and define how large the problem is in regards to hypoendemic areas. It will also help demonstrate that hyper- and holoendemic areas could also achieve elimination with the proper individualized treatment strategies.
4. In Box 1, the last sentence, please define the percentage of treatment coverage meant by “high”. In the letter 65%, 75% and 80% have been discussed. In Box 1 a definite percentage needs to be stated.
5. In the Timeline to achieve target point 3), in addition to resurgence and re-introduction of infection, that spread of infection to previously nonendemic areas should be considered, e.g. as seen for *Wuchereria bancrofti* infection in India (Chand *et al., *2013 ^1^).

I confirm that I have read this submission and believe that I have an appropriate level of expertise to confirm that it is of an acceptable scientific standard.

## References

[1] : Emergence of new foci of filariasis in Madhya Pradesh, India. *Trans R Soc Trop Med Hyg*.2013;107(7) : 10.1093/trstmh/trt040 462-4 10.1093/trstmh/trt040 23687107

# Peer review: Reviewer response for version 1

**DOI:** 10.21956/gatesopenres.14198.r28085

**Published:** 2019-10-22

**Authors:** Johnny Vlaminck

**Affiliations:** 1 Department of Virology, Parasitology and Immunology, Ghent University, Merelbeke, Belgium

I greatly enjoyed reviewing the current letter on the WHO 2030 goals for onchocerciasis as seen from a mathematical modelling perspective. The article read really fluently due to the good language and grammar.

 I found that the letter started by providing a nice overview of the background of onchocerciasis control, the evolution of how modelling assisted in the evaluation of program progress and moving towards an overview of how the 2030 goals differ from the current ones. 

 Next, modelling analysis using the Onchosim and Epioncho models illustrate important insights which are summarized in Box 1.

 The final part of the paper focuses on discussing the practical implications of the current goals and with it, highlights some important research gaps (Table 2).

 I particularly like that it questions the current set stop criteria (Table 2, item 3). I think this is something that is relevant for more than just onchocerciasis. Both diagnostic thresholds and stopping criteria are not set in stone and should be continuously challenged and evaluated using empirical data from field studies supported by models such as Onchosim and Epioncho.

I confirm that I have read this submission and believe that I have an appropriate level of expertise to confirm that it is of an acceptable scientific standard.

# Peer review: Reviewer response for version 1

**DOI:** 10.21956/gatesopenres.14198.r27985

**Published:** 2019-10-22

**Authors:** James A. Whitworth

**Affiliations:** 1 London School of Hygiene & Tropical Medicine, London, UK

This Open Letter is a valuable addition to the scientific literature about onchocerciasis elimination. The authors clearly explain how their modelling work helps to assess the likely progress that can be made using existing approaches to onchocerciasis control, and highlight the scenarios in which these approaches will not achieve the desired goals.

 In some cases the two statistical models used differ in their conclusions, but the authors explain why this is in terms of the underlying assumptions and boundaries of our current knowledge, and thereby outline the limitations of current models. It is encouraging that in most scenarios the models do agree in their conclusions.

 Two aspects were not entirely clear and could be clarified. Firstly, it is not clear to what extent Alternative Treatment Strategies have been modelled, or indeed could be. These are recommended, but while some of these are achievable - increasing coverage and treatment frequency, others, such as adding vector control strategies, moxidectin and yet to be developed macrofilaricide drugs are not so easily implemented. This section of the paper seems rather speculative as currently written.

 It is also not clear whether the comments about Ov16 serology arise from the conclusions of the modelling work; I suspect not. I suppose that the authors are pointing out that uncertainties about the performance of the diagnostic test limits confidence in surveillance and evaluation components of control programmes. But this could be clarified.        

I confirm that I have read this submission and believe that I have an appropriate level of expertise to confirm that it is of an acceptable scientific standard.
